# Development of Ulcerative Colitis in a Patient With Human Immunodeficiency Virus

**DOI:** 10.1155/crgm/2362039

**Published:** 2025-06-18

**Authors:** Clive Jude Miranda, Murad Hayatt Ali, Nariman Hossein-Javaheri, Farhan Azad, Marcellus Anthony Singh, Navpreet Kaur Rana, Nakul Anush Ravish, Thomas Christopher Mahl

**Affiliations:** ^1^Department of Gastroenterology, Hepatology, and Nutrition, State University of New York, Buffalo, New York, USA; ^2^Department of Hematology and Oncology, Inova Fairfax Hospital, Falls Church, Virginia, USA; ^3^Department of Gastroenterology, CHI Health Creighton University Medical Center-Bergan Mercy, Omaha, Nebraska, USA; ^4^Department of Pathology, Hospitals of the University of Pennsylvania, Philadelphia, Pennsylvania, USA

**Keywords:** human immunodeficiency virus, immunology, inflammatory bowel disease

## Abstract

The relationship between the human immunodeficiency virus (HIV) and inflammatory bowel disease (IBD) is poorly understood. The coexistence of the two conditions is uncommon with scattered retrospective studies in the literature. Whereas HIV was initially thought to propagate IBD flares and increase disease severity, more studies are coming out showing that HIV may actually be protective against IBD development and relapse, particularly due to the depletion of CD4 lymphocytes. We present a HIV-positive female with new onset ulcerative colitis at the age of 42. Her HIV was poorly controlled for 25 years but with new gastrointestinal symptoms for 9 months, an endoscopic evaluation was done which revealed a new inflammatory bowel disease diagnosis, which warranted immediate therapy. In describing this patient's case, we discuss the uncommon coexistence of HIV and IBD and investigate potential relationships between the two conditions.

## 1. Introduction

The inflammatory component of inflammatory bowel disease (IBD) is hypothesized to be initiated and perpetuated by an aggressive cell-mediated immune response to an unknown environmental antigen in a genetically susceptible host [1]. Both ulcerative colitis (UC) and Crohn's disease (CD) pathophysiology share similar pathways and result in the intestinal interstitium being infiltrated with large numbers of inflammatory cells, including lymphocytes, neutrophils, and monocytes [2]. The ensuing inflammatory infiltrate results in remarkable destructive processes to tissue architecture including mucosal and transmural injury, goblet cell loss, decreased mucus production, edema, ulcerations, and erosions. All this suggests that leukocytes have an important role in the pathogenesis of IBD with CD4+ T lymphocytes playing particular importance [3]. Human immunodeficiency virus (HIV) can lead to a wide range of clinical manifestations, the most notable being a predisposition to deadly opportunistic infections, primarily due to a depletion in the number of CD4+ T lymphocytes. The biggest obstacle to a cure for HIV is the issue of latently infected cellular reservoirs in memory CD4+ T cells and macrophages [4]. The gastrointestinal tract houses one of these main reservoirs and it is known that chronic gut inflammation is associated with HIV replication, resultant gut-epithelial barrier breakdown, and microbial translocation even in people taking antiretroviral therapy (ART) [5–7].

Despite the breadth of knowledge on the pathophysiology of these two disease entities, the relationship between HIV and IBD remains obscure. There are very few cases in the literature with patients having both these conditions concurrently and, as a result, multiple diverging hypotheses have been proposed to connect the clinical course of IBD in people living with HIV (PLWH). Our case describes a middle-aged female with long-standing HIV who was found to have newly diagnosed UC. In elaborating upon her clinical course, we aim to signify her chronic HIV as being a protective component to the development and progression of IBD.

## 2. Case Presentation

**Figure 1:**
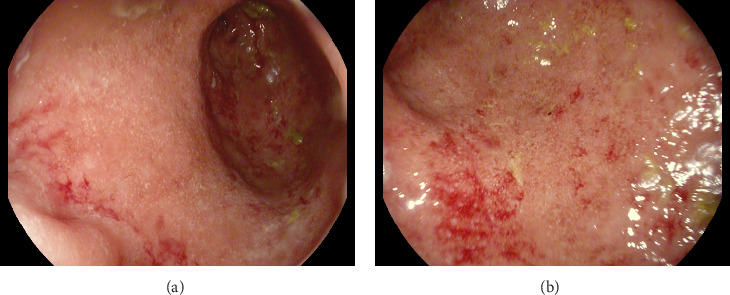
(a) Colonoscopy showing erythematous and friable mucosa in the proximal rectum with scattered diffuse erosions with circumferential involvement noted extending from the anal verge to 15 cm. (b) Colonoscopic view of the distal sigmoid showing aforementioned friable and erythematous mucosa circumferentially with contact oozing and scattered erosions.

**Figure 2:**
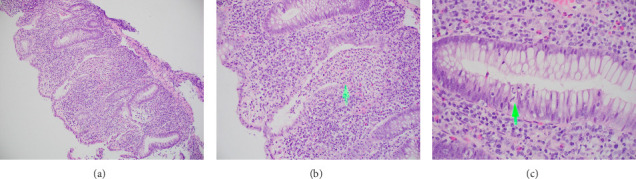
(a) Exuberant mixed inflammatory infiltrate of the lamina propria with overlying mucosal erosion, 100x. (b) Colonic crypt abscess formation (arrow) alongside crypt destruction indicative of ulcerative colitis, 200x. (c) Intraepithelial neutrophilic infiltrate of the colonic crypts (arrow) characteristic for cryptitis, 200x.

A 42-year-old female with a history of HIV diagnosed 25 years ago in India, chronic alcohol use, and depression with a history of suicidal attempts presented for 9 months of abdominal pain, vomiting, and bloody bowel movements mixed with mucous. She reported poor oral intake and an unintentional 18 lb weight loss. The patient was supposed to be on an ART regimen of darunavir, cobicistat, emtricitabine, and tenofovir/alafenamide but was not taking them for approximately 2 years. Workup showed a white count of 4 × 10^9^/L with an absolute neutrophil count of 2 × 10^9^/L. Hemoglobin was at the baseline at 11.6 with lactate at 6.9, AST/ALT 174/114, lipase 432, and stool calprotectin 496. HIV RNA viral load was noted to be 58,500 with an absolute CD4 count of 405, %CD3 cells 85.7, absolute CD3 count 1200, % CD4 cells 28.9, % CD8 cells 55.9, and absolute CD8 count 783. A sexually transmitted disease panel was negative. Colonoscopy showed erythematous and friable mucosa with scattered diffuse erosions with circumferential involvement noted extending from the anal verge to 15 cm (Figures 1(a) and 1(b)). The sigmoid appeared normal past 15 cm. Mayo Endoscopic Score was 3 and Ulcerative Colitis Endoscopic Index of Severity (UCEIS) score was 7, both indicative of severe disease. Rectal pathology showed severe chronic active colitis with cryptitis, crypt abscesses, and mucosal erosions highly indicative of ulcerative colitis (Figures 2(a), 2(b), and 2(c)). Sigmoid pathology was unremarkable. The patient was started on mesalamine enemas with remarkable improvement in symptomatology and discharged.

## 3. Discussion

Our case demonstrates a rare coexistence of HIV and new onset UC. IBD, in and of itself, is an uncommon disease. It follows that, in areas that are highly prevalent with (often untreated) HIV, it is even more difficult to find coexisting IBD in those populations as it is likely these two conditions are simply not prevalent in the same populations. Further evidence of the low prevalence of these two conditions is widely exhibited in medical databases, making the possibility of coexisting HIV and IBD even lower. For example, a 21-year compilation of over 1600 patients between 1988 and 2009 noted only 20 patients with coexisting IBD and HIV [2]. Another 4-year (1989–1993) hospital database of 2 million people revealed 1839 PLWH and 1115 patients with IBD, but only 6 patients harboring both conditions concurrently [8]. A cross-sectional, retrospective, population-based study in Switzerland in 2017 evaluated the frequency of chronic conditions between IBD and non-IBD patients. A total of 1,119,429 patients were studied (4791 of which had IBD) and, although there were large differences in rheumatologic conditions, bone diseases, iron deficiency anemia, and cancer between the two cohorts, there was no statistically significant difference for HIV between the IBD and non-IBD populations [9]. Yen et al., in a nationwide HIV/AIDS patient cohort in Taiwan between 2000–2012, found that IBD had the second highest incidence density in the cohort at 92.24/100,000 person-years [10]. A multicenter, prospective French cohort study spanning 13 years and over 33,400 HIV patients revealed that chronic IBD was one of the most prevalent concurrent autoimmune conditions among the cohort (596.8/100,000 persons) [11]. Demba et al. conducted a retrospective cohort study out of the province of Québec, Canada, to evaluate the incidence and relative risk of autoimmune diseases in HIV-positive patients versus HIV-negative controls. This 2021 study analyzed 4245 HIV-positive patients and 16,493 HIV-negative patients (cumulative mean age of 40 years and 75% male) and showed that there was a strong association of IBD and HIV (adjusted hazard ration: 1.80 and 95% confidence interval: [1.37–2.35]) [12]. Most recently, an unselected nationwide cohort study from Denmark of approximately 9000 HIV-positive patients showed a greater than 2-fold increased risk of IBD compared with matched HIV-negative controls, with further validation in a United States cohort with a higher prevalence of HIV [13]. It is widely accepted that both HIV and IBD are related to immune dysfunction. However, despite the low prevalence of both conditions respectively, does there yet exist a potential pathophysiological relationship between the two? Multiple studies over the years have investigated both the incidence and relapse of IBD in HIV-positive patients versus normal controls, and discussions around this remain nuanced.

Initial theories had proposed that the incidence of IBD is increased in PLWH either due to mucosal inflammation or the loss of CD4 lymphocyte number and function that helps facilitate IBD development (due to their role in facilitating a proinflammatory response) [14, 15]. However, more research has since suggested that the opposite may actually be true and that HIV may be protective against the development or relapse of IBD [8, 15, 16]. A 2016 retrospective study of 58,979 IBD patients (145 of whom were HIV positive) found statistically significant milder IBD disease courses in patients with UC and coexisting HIV [17]. A 7-year study of 121,835 HIV-negative patients with IBD and 475 HIV-positive patients with IBD noted a statistically significant protection against IBD-related obstruction, anemia, and malnutrition in the latter group [18]. In 2010, Viazis et al. studied 20 IBD patients (14 CD and 6 UC) with HIV and 40 HIV-negative IBD patients with the aim of comparing IBD relapse rates per year. The mean relapse rate for the HIV-positive IBD group was 0.016/year of follow-up as compared with 0.053/year of follow-up in the matched controls (*p* < 0.05) with no relapses reported with a CD4 T-lymphocyte count of < 500 [2]. This was pioneering work in furthering the theory that HIV may indeed be protective in IBD development and relapse. The CD4 remission hypothesis postulates that decreased relapse and disease severity in IBD can be seen in HIV, and this is predominantly linked to CD4 T-helper cell depletion, as these CD4 cells (particularly T-helper 1 and 17 subsets) are crucial in facilitating a proinflammatory response through cytokine production and recruitment of macrophages, neutrophils, and other immune cells [19]. Gut-associated lymphoid tissue (GALT) houses over 50% of total circulating T lymphocytes, and it may be that significant GALT T-cell depletion in PLWH may induce mucosal protection in IBD development or relapses in these patients [2, 19]. Our patient had new onset UC at the age of 42. With her absolute CD4 count of 405 despite ART noncompliance, one wonders if this protected her in delaying her first flare for this long. This is an interesting and uncommon case of both conditions coexisting in the same patient, but further research is still needed to investigate any potential immunologic relationship between IBD and HIV. This manuscript was initially presented as an abstract at the Advances in Inflammatory Bowel Disease (AIBD) conference in Orlando, FL, in 2022 [20]. All the patients allowed personal data processing, and informed consent was obtained from all individual participants included in the study.

## Data Availability

The data used to support the findings of this study are included within the article and are also available from the corresponding author upon request. The personal protected patient information used to support the findings of this study are restricted by the University of Buffalo Institutional Review Board in order to protect patient privacy. Data are available from Dr. Clive Jude Miranda at clive.miranda91@gmail.com for researchers who meet the criteria for access to confidential data.
